# Adverse Reactions and Vaccination Preferences Following COVID‐19 and Influenza Vaccination Among Healthcare Workers: A Cross‐Sectional Survey

**DOI:** 10.1002/kjm2.70238

**Published:** 2026-05-13

**Authors:** Yu‐Yin Lin, Yi‐Ling Hsieh, Shih‐Feng Cho, Yun‐Shiuan Chuang, Yun‐Chen Liu, Wen Ku, Chia‐Chi Sung, Yu‐Ling Chang, Chia‐En Hsu, Yi‐Hui Weng, I‐Jui Huang, Chun‐Yu Lin

**Affiliations:** ^1^ Department of Occupational Safety and Health Kaohsiung Medical University Hospital, Kaohsiung Medical University Kaohsiung Taiwan; ^2^ Department of Preventive Medicine Kaohsiung Medical University Hospital, Kaohsiung Medical University Kaohsiung Taiwan; ^3^ Department of Occupational & Environmental Medicine Kaohsiung Medical University Hospital, Kaohsiung Medical University Kaohsiung Taiwan; ^4^ Division of Hematology and Oncology, Department of Internal Medicine Kaohsiung Medical University Hospital, Kaohsiung Medical University Kaohsiung Taiwan; ^5^ Department of Family Medicine Kaohsiung Medical University Hospital, Kaohsiung Medical University Kaohsiung Taiwan; ^6^ Department of Medical Information Kaohsiung Medical University Hospital, Kaohsiung Medical University Kaohsiung Taiwan; ^7^ Division of Infectious Diseases, Department of Internal Medicine Kaohsiung Medical University Hospital, Kaohsiung Medical University Kaohsiung Taiwan; ^8^ School of Medicine, College of Medicine Center for Tropical Medicine and Infectious Diseases, Kaohsiung Medical University Kaohsiung Taiwan

**Keywords:** co‐administration, COVID‐19 vaccine, healthcare professionals, influenza vaccine

## Abstract

Co‐administration of COVID‐19 and influenza vaccines has been promoted to improve coverage, but acceptance among healthcare workers (HCWs) remains uncertain. In total, 1238 participants were recruited. Participants were categorized by vaccination pattern including same‐day co‐administration, non‐concurrent vaccination, or single vaccine. Linear regression and multinomial logistic regression were used to assess adverse reactions, association with prior COVID‐19 vaccination experiences and future vaccination preferences. HCWs who received same‐day co‐administration reported higher adverse reaction scores (1.10 ± 0.92) and prevalence (72.5%) compared to non‐concurrent vaccination (0.88 ± 0.84, 63.0%) or single vaccine recipients (0.68 ± 0.80, 31%–48%). Prior adverse reactions positively correlated with 2024 reactions (*r* = 0.30–0.49, *p* < 0.0001). Non‐concurrent vaccination (*β* = −0.26) and single vaccine receipt (*β* = −0.45) were associated with lower adverse reaction scores than same‐day co‐administration. Among non‐concurrent vaccination recipients, flu‐first vaccination was associated with lower adverse reaction scores than COVID‐first (*β* = 0.30). Past vaccination patterns showed significant association with future choices. In this study, HCWs who received same‐day co‐administration of COVID‐19 and influenza vaccines reported higher adverse reaction scores compared to those who received non‐concurrent or single vaccines. These findings suggest that vaccination scheduling preferences warrant consideration in institutional immunization programs. Prospective research is needed to confirm these associations and determine appropriate vaccination strategies.

## 1 Introduction

Since the COVID‐19 pandemic began in 2020, vaccination has been the cornerstone of public health strategies to control viral spread and reduce severe outcomes. As the pandemic entered a post‐pandemic phase with ongoing variant circulation, many countries, including Taiwan, shifted from emergency campaigns to routine immunization [1, 2]. Taiwan has offered COVID‐19 booster doses since late 2021, and introducing an updated mRNA vaccine targeting Omicron XBB.1.5 in 2023, with policies continuing into 2024 to reduce severe illness and mortality [3, 4]. In autumn and winter 2024, Taiwan CDC promoted co‐administration of COVID‐19 and influenza vaccines to streamline delivery, increase coverage, and address concurrent respiratory virus threats [5]. The Taiwan CDC reported more than 3000 severe influenza cases and more than 200 deaths in the 2023–2024 season, while Omicron XBB subvariants continued to circulate.

Vaccine acceptance is associated with complex psychological and behavioral factors, and vaccination decisions may be influenced by mental health conditions such as anxiety and depression, affecting both intention and uptake [6, 7, 8]. A prior study revealed that vaccination experiences, perceived risks, and health beliefs significantly contribute to hesitancy and could be assessed using health behavior models [9]. Additionally, personal risk assessment and trust in public health policies are critical determinants of COVID‐19 vaccination intention [10]. A systematic review also highlighted additional barriers to influenza vaccination, including perceived health status, credibility of information, quality of health communication, and professional role [11]. These findings suggest that acceptance of co‐administration policies requires careful attention to both individual psychology and broader trust in healthcare systems.

Healthcare workers (HCWs) represent a particularly important population in this context, given both their increased occupational exposure and their role in promoting vaccine confidence. A prospective study by Nguyen et al. reported that HCWs had approximately three times the risk of COVID‐19 infection compared to the general population [12]. Several studies demonstrated that full vaccination significantly reduced infection and hospitalization rates among HCWs [13, 14, 15], while beyond the protective effect, vaccination has also been shown to enhance workforce stability; for example, after vaccination, absenteeism events and missed workdays were significantly reduced [16, 17]. Furthermore, a recent investigation revealed that HCWs who held strong positive beliefs in vaccine efficacy reported lower levels of mental distress during the pandemic [18]. These findings support the importance of vaccination for protecting not only physical health but also the sustainability of healthcare systems.

Recent clinical evidence has shown the feasibility and safety of co‐administering COVID‐19 and influenza vaccines [19, 20, 21, 22, 23]. Co‐administration may enhance spike‐specific antibody responses to SARS‐CoV‐2, suggesting potential immunologic synergy [24]. Moreover, a recent study found no significant difference in adverse events between co‐administration and separate administration [25]. Despite these findings, vaccine acceptance is not completely guaranteed, as concerns regarding safety, perceived risk, and trust in government policies remain influential [26, 27, 28]. In Taiwan, evidence on HCWs' acceptance of and behavior toward COVID‐19 and influenza vaccine co‐administration remains limited; accordingly, we examined whether prior adverse reactions to COVID‐19 vaccination (2021–2022) and real‐world experiences during the 2024 campaign, particularly vaccination timing and sequence, were associated with HCWs' future vaccination preferences.

## 2 Methods

### 2.1 Study Design and Participants

This was a single‐center, cross‐sectional study using a questionnaire completed by participants who voluntarily enrolled. Between January 20 and February 20, 2025, HCWs aged ≥ 20 years with prior COVID‐19 vaccination experience (2021–2022) who had received COVID‐19 and/or influenza vaccines in 2024 were recruited. Participants were categorized into four vaccination patterns based on their 2024 vaccination patterns: (1) receipt of both COVID‐19 and influenza vaccines on the same day; (2) non‐concurrent vaccination protocols; (3) receipt of influenza vaccine only; or (4) receipt of COVID‐19 vaccine only. All participants provided informed consent before participation. This study was conducted in accordance with the Declaration of Helsinki and approved by the Institutional Review Board of Kaohsiung Medical University (KMUHIRB‐E(II)‐20250014).

### 2.2 Questionnaire

An electronic questionnaire with conditional branching logic was administered to ensure response coherence and data completeness. The questionnaire comprised eight sections: (1) demographic information (year of birth and employment commencement); (2) prior adverse reactions to COVID‐19 vaccination in 2021/2022; (3) 2024 vaccination status (concurrent, sequential, or single‐vaccine receipt); (4) adverse reactions following same‐day co‐administration of both vaccines; (5) vaccination sequence and adverse reactions among those receiving non‐concurrent vaccination; (6) adverse reactions among influenza vaccine‐only recipients; (7) adverse reactions among COVID‐19 vaccine‐only recipients; and (8) future vaccination preferences and institutional service suggestions. For the assessment of adverse reactions, a 6‐point symptom‐duration scale [29] was used to quantify participants' post‐vaccination experiences. The types of adverse reactions included: fever (body temperature > 37.5°C), chills, significant fatigue, headache, joint pain, muscle soreness, and other local or systemic discomforts. Participants rated the duration of each symptom as follows: 0 = “*No discomfort*,” 1 = “*Mild*” (duration < 24 h), 2 = “*Slight*” (duration 24–48 h), 3 = “*Moderate*” (duration 48–72 h), 4 = “*Severe*” (duration 72–96 h), and 5 = “*Very severe*” (duration > 96 h). This approach was intended to enhance the quantification of subjective experiences and facilitate comparability in subsequent analysis.

To examine associations between past experiences and current vaccination behaviors, the questionnaire assessed participants' COVID‐19 vaccination history (2021–2022), including prior adverse reactions, and their 2024 vaccination patterns (same‐day co‐administration, non‐concurrent vaccination, or single‐vaccine receipt).

### 2.3 Statistical Analysis

Demographic characteristics are expressed as count (percentages) or mean ± standard deviations. The differences in categorical or continuous variables among participants who received influenza and COVID‐19 vaccines on the same day, separate days, or received only a single vaccine were analyzed using the chi‐square test, Student's *t*‐test, or analysis of variance (ANOVA), as appropriate, while post hoc analysis was performed using the Scheffé test if significant differences were found.

The correlation between adverse reactions from COVID‐19 vaccination in 2021 and vaccination in 2024 was determined using Pearson correlation, with univariable and multivariable linear regression used to evaluate the relationship between adverse reactions and the mode of influenza and COVID‐19 vaccination. In the multivariable model, adverse reactions reported in 2021 were included as an adjustment variable. Univariable and multivariable multinomial logistic regression models were used to identify factors associated with the mode of vaccination in 2024 and 2025. Adverse reactions reported in 2021 were included as an adjustment variable in the multivariable models. In these regressions, Model 1 was a crude model without any adjustments while Model 2 was the adjusted model. All statistical analyzes were performed using SAS 9.4 statistical software. Results with *p* < 0.05 were considered statistically significant.

## 3 Results

A total of 1238 participants were enrolled, comprising 440 (35.5%) who received both vaccines on the same day, 211 (17.0%) who received non‐concurrent vaccination, 580 (46.8%) who received the influenza vaccine only, and 7 (0.6%) who received the COVID‐19 vaccine only. Baseline characteristics were comparable across groups, with mean ages of 43–45 years (*p* = 0.0904) and 16–17 years of employment (*p* = 0.2264). However, significant differences emerged in adverse reaction patterns. Prior COVID‐19 vaccination reactions in 2021 were less severe among same‐day recipients than non‐concurrent vaccination or single‐vaccine recipients (*p* = 0.0005). Similarly, post‐2024 vaccination adverse reactions were most common among same‐day recipients (72.5%), followed by non‐concurrent vaccination (63.0%) and single‐vaccine recipients (31%–48%, *p* < 0.0001), with post hoc tests showing significant pairwise differences (Table 1).

**TABLE 1:** The baseline characteristics of the study population.

	Flu/COVID vaccination (*n* = 1238)			*p*	Significant in post hoc
	Group 1: Both, same‐day (*n* = 440)	Group 2: Both, non‐concurrent (*n* = 211)	Group 3: Flu only or COVID only (*n* = 587)		
Age (years)	43.95 ± 9.90	45.34 ± 9.36	43.59 ± 9.87	0.0904	
≤ 45	232 (54.70%)	106 (52.50%)	316 (55.30%)	0.7802	
> 45	192 (45.30%)	96 (47.50%)	255 (44.70%)		
Working years	16.04 ± 11.33	17.43 ± 11.20	15.95 ± 10.85	0.2264	
≤ 15	236 (53.60%)	96 (45.50%)	300 (51.10%)	0.1509	
> 15	204 (46.40%)	115 (54.50%)	287 (48.90%)		
Side effects of prior COVID vaccination in 2021					
Scores (Mean ± SD (median, IQR))	1.58 ± 1.09 (2, 1–2)	1.84 ± 1.11 (2, 1–3)	1.83 ± 1.12 (2, 1–3)	0.0005	1 versus 2, 1 versus 3
Severity				0.0016	
No	77 (17.50%)	20 (9.50%)	63 (10.70%)		
Yes	363 (82.50%)	191 (90.50%)	524 (89.30%)		
Minimal	134	66	174		
Mild	152	68	202		
Moderate	60	48	110		
Severe	10	2	26		
Very severe	7	7	12		
Side effects after vaccination in 2024					
Scores (Mean ± SD (median, IQR))	1.10 ± 0.92 (1, 0–2)	0.88 ± 0.84 (1, 0–1)	0.68 ± 0.80 (1, 0–1)	< 0.0001	1 versus 2, 1 versus 3, 2 versus 3
Side effects				< 0.0001	
No	121 (27.50%)	78 (37.00%)	284 (48.40%)		
Yes	319 (72.50%)	133 (63.00%)	303 (51.60%)		
Minimal	192	91	226		
Mild	96	32	62		
Moderate	26	10	12		
Severe	3	0	1		
Very severe	2	0	2		
Future vaccination patterns				< 0.0001	
Both, same‐day	381 (86.60%)	37 (17.50%)	38 (6.50%)		
Both, non‐concurrent	30 (6.80%)	123 (58.30%)	86 (14.70%)		
Flu only	28 (6.40%)	51 (24.20%)	462 (78.70%)		
COVID only	1 (0.20%)	0	1 (0.20%)		

Abbreviations: COVID, COVID‐19; Flu, Influenza; IQR, interquartile range.

Table 2 presents comparisons stratified by vaccination patterns. Among the 211 participants who received non‐concurrent vaccination, no significant differences in demographics or prior 2021 COVID‐19 vaccination adverse reactions were observed between flu‐first (*n* = 136) and COVID‐first recipients (*n* = 75). However, COVID‐first recipients experienced significantly higher adverse reaction scores following 2024 vaccination (1.05 ± 0.93) compared to flu‐first recipients (0.78 ± 0.77, *p* = 0.0224), although the overall prevalence of adverse reactions did not differ significantly between groups (69.3% vs. 59.6%, *p* = 0.1592).

**TABLE 2:** Comparison of characteristics and adverse reactions across vaccination groups.

	Both, non‐concurrent (*n* = 211)		*p*	COVID only or Flu only (*n* = 587)		*p*	Both Flu and COVID vaccination (*n* = 651)		*p*
	Flu‐first (*n* = 136)	COVID‐first (*n* = 75)		Flu only (*n* = 580)	COVID only (*n* = 7)		Both, same‐day (*n* = 440)	Both, non‐concurrent (*n* = 211)	
Age (years)	45.81 ± 8.42	44.52 ± 10.85	0.3836	43.74 ± 9.77	30.86 ± 10.62	0.0021	43.95 ± 9.90	45.34 ± 9.36	0.0938
≤ 45	63 (48.80%)	43 (58.90%)	0.1687	310 (55.00%)	6 (85.70%)	0.1377	232 (54.70%)	106 (52.50%)	0.5988
> 45	66 (51.20%)	30 (41.10%)		254 (45.00%)	1 (14.30%)		192 (45.30%)	96 (47.50%)	
Working years	18.34 ± 11.04	15.77 ± 11.35	0.7716	16.07 ± 10.79	6.14 ± 12.29	0.0055	16.04 ± 11.33	17.43 ± 11.20	0.1424
≤ 15	59 (43.40%)	37 (49.30%)	0.4060	294 (50.70%)	6 (85.70%)	0.1234	236 (53.60%)	96 (45.50%)	0.0519
> 15	77 (56.60%)	38 (50.70%)		286 (49.30%)	1 (14.30%)		204 (46.40%)	77 (56.60%)	
Side effects of prior COVID vaccination in 2021									
Scores (Mean ± SD (median, IQR))	1.96 ± 1.15 (2, 1–3)	1.64 ± 1.01 (2, 1–2)	0.2028	1.83 ± 1.12 (2, 1–3)	1.29 ± 1.11 (1, 0–2)	0.2246	1.58 ± 1.09 (2, 1–2)	1.84 ± 1.11 (2, 1–3)	0.0037
Severity			0.0561			0.1674			0.0071
No	9 (6.60%)	11 (14.70%)		61 (10.50%)	2 (28.60%)		77 (17.50%)	20 (9.50%)	
Yes	127 (93.40%)	64 (85.30%)		519 (89.50%)	5 (71.40%)		363 (82.50%)	191 (90.50%)	
Minimal	43	23		172	2		134	66	
Mild	45	23		200	2		152	68	
Moderate	30	18		109	1		60	48	
Severe	2	0		26	0		10	2	
Very severe	7	0		12	0		7	7	
Side effects after vaccination in 2024									
Scores (Mean ± SD (median, IQR))	0.78 ± 0.77 (1, 0–1)	1.05 ± 0.93 (1, 0–2)	0.0224	0.67 ± 0.79 (1, 0–1)	1.29 ± 1.38 (1, 0–3)	0.2521	1.10 ± 0.92 (1, 0–2)	0.88 ± 0.84 (1, 0–1)	0.0031
Side effects			0.1592			1			0.0141
No	55 (40.40%)	23 (30.70%)		281 (48.40%)	3 (42.90%)		121 (27.50%)	78 (37.00%)	
Yes	81 (59.60%)	52 (69.30%)		299 (51.60%)	4 (57.10%)		319 (72.50%)	133 (63.00%)	
Minimal	59	32		225	1		192	91	
Mild	19	13		61	1		96	32	
Moderate	3	7		10	2		26	10	
Severe	0	0		1	0		3	0	
Very severe	0	0		2	0		2	0	
Future vaccination patterns			0.6541			0.0003			< 0.0001
Both, same‐day	26 (19.10%)	11 (14.70%)		36 (6.20%)	2 (28.60%)		381 (86.60%)	37 (17.50%)	
Both, non‐concurrent	79 (58.10%)	44 (58.70%)		84 (14.50%)	2 (28.60%)		30 (6.80%)	123 (58.30%)	
Flu only	31 (22.80%)	20 (26.70%)		460 (79.30%)	2 (28.60%)		28 (6.40%)	51 (24.20%)	
COVID only	0	0		0	1 (14.30%)		1 (0.20%)	0	

*Note:* Flu‐first: participants who received influenza vaccine before COVID‐19 vaccination.

Abbreviations: COVID, COVID‐19; Flu, influenza; IQR, interquartile range.

Among single‐vaccine recipients (*n* = 587), COVID‐only recipients (*n* = 7) were significantly younger (30.86 ± 10.62 vs. 43.74 ± 9.77 years, *p* = 0.0021) and had fewer years of employment (6.14 ± 12.29 vs. 16.07 ± 10.79 years, *p* = 0.0055) compared to flu‐only recipients (*n* = 580). No significant differences were observed in adverse reactions between these groups.

Among the 651 participants who received both vaccines, same‐day and non‐concurrent vaccination recipients had similar demographic profiles. However, those who had received same‐day co‐administration (*n* = 440) experienced significantly lower adverse reaction scores from prior 2021 COVID‐19 vaccination (1.58 ± 1.09) compared to those who chose non‐concurrent vaccination (*n* = 211, 1.84 ± 1.11, *p* = 0.0037), with corresponding differences in severity distribution (*p* = 0.0071). Conversely, following 2024 vaccination, same‐day recipients reported significantly higher adverse reaction scores (1.10 ± 0.92 vs. 0.88 ± 0.84, *p* = 0.0031) and higher prevalence of any adverse reactions (72.5% vs. 63.0%, *p* = 0.0141).

Table 3 demonstrates significant positive correlations between 2021 and 2024 adverse reaction scores across non‐concurrent vaccination groups, with the moderate correlation observed among dual‐vaccine recipients (*r* = 0.49) compared to single‐vaccine recipients (*r* = 0.30) or all participants combined (*r* = 0.38). Age showed no correlation with adverse reaction severity in any group. These results suggest that prior adverse reaction experience is significantly associated with reporting of subsequent adverse reactions, particularly among those receiving both vaccines.

**TABLE 3:** The correlation between side effects in 2021 and in 2024 in different groups.

	All participants (*n* = 1238)	Both flu and COVID vaccination (*n* = 651)	Flu only or COVID only (*n* = 587)
	Side effect scores (2021)		
Age	−0.03	−0.04	−0.02
Side effect scores (2024)	0.38**	0.49**	0.30**

Abbreviations: COVID, COVID‐19; Flu, influenza.

** *p* < 0.0001.

**FIGURE 1:**
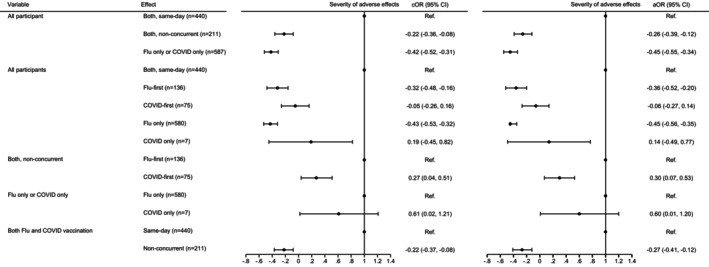
The association between vaccination pattern and severity of adverse effects in 2024. Abbreviations: aOR, adjusted odds ratio; cOR, crude odds ratio; COVID, COVID‐19; Flu, Influenza.

The estimates and confidence intervals of vaccination pattern and severity of adverse effects in 2024 are illustrated in a forest plot (Figure 1). Compared to same‐day co‐administration, both non‐concurrent vaccination (adjusted *β* = −0.26, 95% CI: −0.39, −0.12) and single‐vaccine receipt (adjusted *β* = −0.45, 95% CI: −0.55, −0.34) were associated with significantly lower adverse reaction scores. Stratified analyzes revealed that flu‐first vaccination was associated with significantly lower scores (adjusted *β* = −0.36, 95% CI: −0.52, −0.20), while COVID‐first and COVID‐only vaccination showed no significant differences. Flu‐only vaccination was associated with significantly lower scores (adjusted *β* = −0.45, 95% CI: −0.56, −0.35). Among those receiving non‐concurrent vaccination, COVID‐first vaccination was associated with significantly higher scores than flu‐first vaccination (adjusted *β* = 0.30, 95% CI: 0.07, 0.53). Among those who received both vaccines, non‐concurrent vaccination remained protective (adjusted *β* = −0.27, 95% CI: −0.41, −0.12).

Table 4 presents the association between past vaccination pattern and future preferences. Compared to same‐day co‐administration, flu‐first vaccination had higher odds of preferring future non‐concurrent vaccination (adjusted OR = 2.46, 95% CI: 2.44, 2.49), with similar patterns observed for COVID‐first vaccination (adjusted OR = 2.53, 95% CI: 2.50, 2.56). Flu‐only vaccination showed no significant association with non‐concurrent vaccination preference (adjusted OR = 1.00, 95% CI: 0.99, 1.00) but had higher odds of continuing flu‐only vaccination (adjusted OR = 1.77, 95% CI: 1.77, 1.78). COVID‐only vaccination showed no significant associations due to limited sample size.

**TABLE 4:** Influenza and COVID‐19 vaccination patterns and preferences for future co‐administration.

	Model 1			Model 2		
	Both, non‐concurrent	Flu only	COVID only	Both, non‐concurrent	Flu only	COVID only
All participants (*n* = 1238)						
Both, same‐day (*n* = 440)	1	1	1	1	1	1
Both, non‐concurrent (*n* = 211)	42.22 (25.03, 71.20)	18.76 (10.59, 33.21)	—	41.95 (24.84, 70.84)	18.26 (10.30, 32.37)	—
Flu only or COVID only (*n* = 587)	28.74 (16.87, 48.97)	165.43 (99.68, 274.57)	10.03 (0.62, 163.53)	28.64 (16.80, 48.81)	162.97 (98.17, 270.57)	10.18 (0.62, 166.84)
All participants (*n* = 1238)						
Both, same‐day (*n* = 440)	1	1	1	1	1	1
Flu‐first (*n* = 136)	2.46 (2.44, 2.48)	0.84 (0.83, 0.84)	0.69 (0.60, 0.78)	2.46 (2.44, 2.49)	0.83 (0.83, 0.84)	0.69 (0.61, 0.79)
COVID‐first (*n* = 75)	2.53 (2.50, 2.56)	0.89 (0.88, 0.90)	0.70 (0.58, 0.83)	2.53 (2.50, 2.56)	0.89 (0.88, 0.90)	0.70 (0.59, 0.84)
Flu only (*n* = 580)	0.99 (0.99, 1.00)	1.77 (1.77, 1.78)	0.73 (0.68, 0.78)	1.00 (0.99, 1.00)	1.77 (1.77, 1.78)	0.74 (0.69, 0.78)
COVID only (*n* = 7)	—	—	—	—	—	—
Both, non‐concurrent (*n* = 211)						
Flu‐first (*n* = 136)	1	1	1	1	1	1
COVID‐first (*n* = 75)	1.32 (0.59, 2.92)	1.53 (0.62, 3.76)	—	1.31 (0.59, 2.90)	1.54 (0.62, 3.79)	—
Flu only or COVID only (*n* = 587)						
Flu only (*n* = 580)	1	1	1	1	1	1
COVID only (*n* = 7)	0.43 (0.06, 3.16)	0.08 (0.01, 0.57)	—	0.42 (0.06, 3.12)	0.08 (0.01, 0.56)	—
Both flu and COVID vaccination (*n* = 651)						
Same‐day (*n* = 440)	1	1	1	1	1	1
Non‐concurrent (*n* = 211)	42.22 (25.03, 71.20)	18.76 (10.59, 33.21)	—	42.31 (25.01, 71.57)	18.28 (10.30, 32.44)	—

*Note:* Preferences for future co‐administration, used both, same‐day as reference group. Model 1: Crude, Model 2: adjusted to the appearance of prior side effects.

Abbreviations: aOR, adjusted odds ratio; cOR, crude odds ratio; COVID, COVID‐19; Flu, influenza.

Within the non‐concurrent vaccination group, compared to flu‐first vaccination, COVID‐first vaccination had no significant association with future preferences for non‐concurrent vaccination (adjusted OR = 1.31, 95% CI: 0.59, 2.90) or flu‐only vaccination (adjusted OR = 1.54, 95% CI: 0.62, 3.79). Within single vaccine recipients, COVID‐only vaccination showed no significant association (adjusted OR = 0.08, 95% CI: 0.01, 0.56) compared to flu‐only vaccination.

Among those who received both vaccines, non‐concurrent vaccination had markedly higher odds of future preference for repeating this approach compared to same‐day co‐administration (adjusted OR = 42.31, 95% CI: 25.01, 71.57). Adjustment for prior adverse reactions produced minimal changes in odds ratios compared to crude models, indicating that past vaccination behavior rather than adverse reaction experience was the primary driver of future vaccination preferences, demonstrating strong behavioral persistence.

## 4 Discussion

The present study revealed significant associations between vaccination pattern and adverse reactions. Same‐day co‐administration was associated with higher adverse reaction incidence (72.5%) and severity compared to non‐concurrent vaccination (63.0%) or single‐vaccine receipt (31%–48%). Notably, prior COVID‐19 vaccination reactions (2021–2022) showed moderate associations with 2024 responses (*r* = 0.38–0.49, *p* < 0.0001), indicating that individuals with prior adverse reactions were more likely to report subsequent reactions.

This finding supports the results of previous studies that co‐administration can effectively boost anti‐viral immunity [20, 30, 31], but related adverse reactions were also observed. In addition, the participants who received the vaccines on separate days—especially when the influenza vaccine was administered before the COVID‐19 vaccine—experienced a lower severity of adverse reactions. These results suggest that the vaccination patterns are associated with differences in adverse events and should be carefully considered when planning immunization strategies.

Despite the higher frequency of reported adverse reactions, co‐administration remains operationally feasible. However, adverse reactions were more frequently reported with same‐day co‐administration and in the COVID‐19–only group, warranting attention. Although mechanistic data were not directly assessed in this study, the stronger immune stimulation elicited by COVID‐19 vaccination, alone or combined with influenza vaccination, may partly explain the higher reactogenicity observed [22, 32]. A previous study revealed that certain professional categories, like nurses and administrative staff, are less willing to receive coadministration of COVID‐19/influenza vaccines [33].

The present study differs from our prior work in both methodology and respondent profiles. We used a long‐standing in‐hospital mail‐based survey system, which consistently yields a nursing‐dominant sample. Given nurses' irregular schedules and circadian disruption, they might prefer time‐efficient vaccination options while weighing tolerance of adverse reactions. These findings support tailoring vaccination scheduling, strengthening education for hesitant staff, and incorporating flexible strategies into occupational health policies to optimize safety and uptake.

Several limitations should be noted. Firstly, the cross‐sectional design precludes causal inference, so we could not determine whether vaccination patterns caused specific reactions or if anticipated reactions led individuals to choose specific strategies. Furthermore, our sample included only vaccinated HCWs at a single center. Notably, gender data were not collected to maintain anonymity, and although prior institutional data suggested that more than 80% of the staff were female, the gender distribution in this sample could not be confirmed [18, 34]. Some key potential confounders, including prior COVID‐19 infection, underlying comorbidities and individual‐level vaccination intervals, were not collected so the absence of these variables might have introduced residual confounding possibly leading to over‐ or underestimation of the observed associations. Secondly, small subgroup sizes affected statistical precision, as in the COVID‐19–only group (*n* = 7) being significantly smaller than the flu‐only group (*n* = 580), resulting in wide confidence intervals and reduced statistical power in regression analyzes. Thirdly, reliance on self‐reporting measures might introduce recall and measurement bias with participants recalling reactions from 2 to 3 years previously, which are subject to memory decay. Finally, our symptom‐duration scale is subjective and unvalidated, potentially leading to variability in severity interpretation.

While these limitations could affect causal interpretation and precision, they do not negate the consistency and practical relevance of the observed associations. Although 2024 institutional administrative data could not be directly linked to individual adverse outcomes and were not included in regression models, they provided contextual information for interpreting the observed associations by offering real‐world insight into vaccination practices. In particular, the predominance of short‐interval schedules suggested relatively limited variability in exposure timing, which might help constrain interval‐related heterogeneity (Table S1); however, the inability to link these data to individual outcomes prevented formal adjustment in regression analyzes. Taken together, these limitations should be considered when interpreting the findings.

Beyond these limitations, our findings contribute to the literature in several complementary ways. Using a relatively large real‐world cohort of HCWs, we extend beyond conventional comparisons of co‐administration versus separate vaccination by examining vaccination sequence within non‐concurrent strategies, showing that an influenza‐first approach is associated with lower reactogenicity and highlighting vaccination order as a clinically relevant yet underexplored factor. In addition, by linking prior COVID‐19 vaccination experiences (2021–2022) with subsequent reactions in 2024, we demonstrate consistent correlations that support the concept of persistent individual reactogenicity patterns. Furthermore, our analysis incorporates behavioral outcomes, showing that prior vaccination patterns strongly predict future preferences independent of adverse reaction severity, suggesting that vaccination decisions may be driven more by behavioral persistence than by prior adverse experiences. Finally, our results provide real‐world evidence that differs from some clinical trial findings, particularly regarding the higher reactogenicity observed with same‐day co‐administration, underscoring the importance of population‐specific and implementation‐context considerations.

In conclusion, our findings have practical implications for vaccination strategies in healthcare settings. Same‐day co‐administration was associated with higher reactogenicity, suggesting that supportive measures such as flexible scheduling or post‐vaccination rest might be warranted. Non‐concurrent vaccination, particularly with an influenza‐first sequence, could reduce adverse reactions, and while maintaining high vaccine coverage remains essential, optimizing vaccination scheduling could improve both tolerability and acceptance among HCWs.

## Funding

This work was supported in part by grants from Kaohsiung Medical University Hospital (KMUH113‐3M15) and Kaohsiung Medical University Research Center Grant (KMU‐TC114B01). The funders played no role in the design, interpretation or writing of the manuscript.

## Conflicts of Interest

The authors declare no conflicts of interest.

## Supporting information


**Table S1:** Distribution of vaccination patterns and intervals derived from 2024 institutional administrative data (*N* = 2132).

## Data Availability

The data that support the findings of this study are available from the corresponding author upon reasonable request.
