# Supplementary material for: Adverse Reactions and Vaccination Preferences Following COVID‐19 and Influenza Vaccination Among Healthcare Workers: A Cross‐Sectional Survey
Source: Kaohsiung J Med Sci. 2026 May 13:e70238. Online ahead of print. doi: 10.1002/kjm2.70238 (PMC13399778; doi:10.1002/kjm2.70238)
Supplement: Supplementary file 1 — Table S1: Distribution of vaccination patterns and intervals derived from 2024 institutional administrative data (N = 2132). [file KJM2-9999-e70238-s001.docx]

**Table S1. Distribution of vaccination patterns and intervals derived from 2024 institutional administrative data (N = 2,132)**

| **Category** | **n** | **% of total** | **% within subgroup** |
| --- | --- | --- | --- |
| **Vaccination pattern** |  |  |  |
| Same-day vaccination | 1,592 | 74.7 | – |
| Non-concurrent vaccination | 540 | 25.3 | 100 |
| **Sequence (non-concurrent only)** |  |  |  |
| Flu-first | 518 | 24.3 | 95.9 |
| COVID-first | 22 | 1 | 4.1 |
| **Interval (Flu-first)** |  |  |  |
| ≤10 days | 98 | 4.6 | 18.9 |
| 11–20 days | 108 | 5.1 | 20.8 |
| 21–30 days | 22 | 1 | 4.2 |
| 31–60 days | 27 | 1.3 | 5.2 |
| 61–90 days | 12 | 0.6 | 2.3 |
| ≥91 days | 251 | 11.8 | 48.5 |
| **Interval (COVID-first)** |  |  |  |
| ≤10 days | 13 | 0.6 | 59.1 |
| 11–20 days | 2 | 0.1 | 9.1 |
| 21–30 days | 0 | 0 | 0 |
| 31–60 days | 3 | 0.1 | 13.6 |
| 61–90 days | 3 | 0.1 | 13.6 |
| ≥91 days | 1 | 0 | 4.5 |
